# Does Ras/Rho Have Skin in the Game: The Importance of the Isoprenoid Biosynthesis Pathway in Merkel Cell Carcinoma Cell Lines

**DOI:** 10.3390/cancers18101579

**Published:** 2026-05-13

**Authors:** Louise N. Blaha, Nicole M. Derosia, Jeffrey D. Neighbors, Raymond J. Hohl

**Affiliations:** 1 Penn State Cancer Institute, Hershey, PA 17033, USA; lblaha@pennstatehealth.psu.edu (L.N.B.); nderosia@pennstatehealth.psu.edu (N.M.D.); jneighbors@pennstatehealth.psu.edu (J.D.N.); 2 Department of Molecular and Precision Medicine, Penn State College of Medicine, Hershey, PA 17033, USA; 3 Department of Medicine, Penn State College of Medicine, Hershey, PA 17033, USA

**Keywords:** Merkel cell carcinoma, isoprenoid biosynthesis pathway, statins, Ras, Rho, farnesylation, geranylgeranylation

## Abstract

Background: Merkel cell carcinoma (MCC) is a rare malignancy of the skin caused by Merkel cell polyomavirus (80% of cases) or by ultraviolet (UV) sun exposure (20% of cases). The isoprenoid biosynthesis pathway (IBP) is an essential metabolic pathway shown to be upregulated in tumorigenesis, causing aberrant activation of Ras and Rho GTPases and resulting in unregulated cellular proliferation and survival. Methods: Through the use of pharmacological inhibitors of the IBP, we assessed the role of the IBP and its downstream targets in viral-positive and viral-negative MCC cell lines. To identify the most critical IBP intermediates, cellular metabolic activity, cell death and cell cycle distribution were measured after IBP perturbation. Results: Across all cell lines, treatment with IBP inhibitors, especially fluvastatin, decreased metabolic activity; however, perturbation of different intermediates resulted in variable responses between viral-positive and viral-negative cell lines. Conclusions: Our findings demonstrate varying dependence on the IBP between viral-positive and viral-negative MCC and highlight the importance of cellular dynamics when determining a treatment regimen for patients with MCC.

## Simple Summary

Statins are cholesterol-lowering agents that have also been shown to affect how cancer cells grow and survive. Statins target a biochemical pathway called the isoprenoid biosynthesis pathway (IBP), which has been shown to be an ideal target for therapeutic intervention. In our work, we aim to determine whether targeting the IBP through statins and other drugs is an effective way to disrupt carcinogenic behaviors in Merkel cell carcinoma cells. Our results show that cells without Merkel cell polyomavirus infection rely more heavily on this pathway, suggesting that viral and non-viral Merkel cell carcinoma should be treated as distinct diseases. This study provides insight into how underlying biology could impact treatment response, with the hopes of improving both future therapeutic strategies and outcomes for patients.

## 1. Introduction

Merkel cell carcinoma (MCC) is a rare but deadly cutaneous malignancy that predominantly affects the elderly population (>75 years) and, occasionally, younger immunosuppressed individuals [1]. In 80% of cases, MCC development can be attributed to the presence of Merkel cell polyomavirus (MCPyV), while the remaining 20% of cases result from excessive UV exposure [2]. In the US, the incidence of MCC has shown a steady upward trend, with 2000 new cases diagnosed annually, and a 93% increase between 2000 and 2013 [3]. First-line treatment for MCC typically involves surgical tumor excision with sentinel lymph node biopsy, in addition to radiotherapy in cases where surgery may not be feasible or may be harmful. These approaches can be combined with systemic therapies such as chemotherapy (e.g., etoposide, cisplatin) or immunotherapy with anti-PD-L1 agents; however, chemotherapy has shown limited durability of response. While current treatment strategies using anti-PD-1/PD-L1 immune checkpoint inhibitors (e.g., avelumab, pembrolizumab) have extended life expectancy for patients with MCC, five-year overall survival rates range from 35.2 to 82.9%; therefore, there remains a pressing need for alternative therapeutic options [4,5,6,7].

VN-MCC is associated with UV exposure and a high tumor mutational burden, while VP-MCC is driven by integration of MCPyV into the host genome and exhibits a low mutational burden. Despite these distinct etiologies, both subtypes frequently harbor alterations in PI3K–AKT–mTOR and RAS signaling, including activating mutations in AKT1, HRAS, and PIK3CA and loss-of-function mutations in PTEN, NF1, and TSC1 [2]. These pathways are dependent on the IBP for the production of farnesyl and geranylgeranyl intermediates required for small-GTPase prenylation and membrane localization. While distinct in their underlying mechanisms, these overlapping mutational profiles indicate a shared metabolic dependency that could be therapeutically targeted in MCC regardless of viral status.

**Figure 1:**
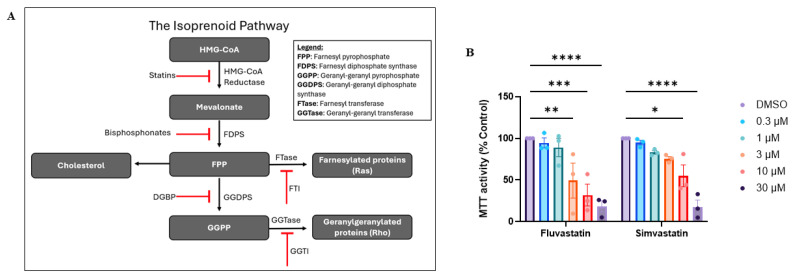
Targeting the isoprenoid biosynthesis pathway (IBP) with inhibitors depletes essential isoprenoid products. (**A**) Schematic of the IBP demonstrating inhibitors and their target enzymes at each step of the pathway. (**B**) Investigation of the effects of several statins on cellular viability in a viral-negative (MCC13) MCC line. Data points represent three individual experiments (n = 3), each with three technical replicates, and were calculated as a percentage of the control. Error bars indicate mean values ± SEM. * *p* < 0.05, ** *p* < 0.01, *** *p* < 0.001, and **** *p* < 0.0001.

Statins are one of the most commonly used medications in the U.S. [8], targeting the enzyme HMG-CoA reductase (HMGCR) in the isoprenoid biosynthesis pathway (IBP) to reduce cholesterol levels in cases of hypercholesterolemia (Figure 1A). More recently, statins have been studied for repurposing as anti-cancer agents, as IBP inhibition can reduce tumor progression through processes such as decreased cell proliferation and invasion [9]. The IBP, or mevalonate pathway, is an essential metabolic pathway that converts acetyl-CoA into several prenyl pyrophosphate molecules, which act as precursors for the formation of sterols, dolichols, diterpenes, and retinol [10], in addition to serving as substrates for the post-translational prenylation of signaling proteins [11]. IBP intermediates farnesyl pyrophosphate (FPP) and geranylgeranyl pyrophosphate (GGPP) mediate the prenylation of Ras and Rho GTPases, respectively, in which farnesyl or geranylgeranyl moieties are covalently attached to proteins to localize them to membranes for signal transduction. In physiological contexts, farnesylation and geranylgeranylation are required for proper intracellular localization of GTPases and are tightly regulated processes. However, in cancer, increased IBP flux can amplify prenylation, thereby promoting increased Ras and Rho GTPase activity and enhancing oncogenic cellular behavior and tumor metastasis [12].

In this study, we characterize the involvement of the IBP in MCC by assessing how inhibition of this key pathway impacts pro-tumorigenic behaviors in viral-negative MCC (VN-MCC) and viral-positive MCC (VP-MCC) cell lines. Using pharmacological inhibitors of the IBP (statins, bisphosphonates, and prenyl transferase inhibitors), we evaluate the effects of pathway perturbation on cellular viability, cell-cycle distribution, apoptosis, and protein expression through analysis of downstream prenylated proteins. Our findings reveal distinct responses between VN-MCC and VP-MCC cells, with VN-MCC13 cells exhibiting greater sensitivity to geranylgeranylation inhibition. Changes in Rho (Cdc42, Rac1, and RhoA) mRNA and protein levels, along with viability, are rescued by GGPP addback, suggesting geranylgeranylation as the primary mediator of the observed phenotypes. While statins have been widely investigated as anti-cancer agents in other malignancies, their effects in MCC remain unexplored. Our work addresses this gap by evaluating the ability of statins to perturb tumorigenic phenotypes in MCC cell lines, in addition to characterizing the differential effects of IBP modulation on VP-MCC and VN-MCC, thereby providing novel insight into potential therapeutic avenues for this aggressive cancer.

## 2. Materials and Methods

### 2.1. Cell Culture

MKL-1 (viral-positive) and MCC13 (viral-negative) human Merkel cell carcinoma cell lines were obtained from the European Collection of Authenticated Cell Cultures (ECACC; Salisbury, UK; Cat# 09111801 and 10092302). Both cell lines were cultured in RPMI-1640 medium supplemented with 10% fetal bovine serum for MKL-1 cells and 15% fetal bovine serum for MCC13 cells. Cells were kept at 37 °C at 5% CO_2_ and passaged upon reaching 80% confluency. Cells were not used beyond passage 25.

### 2.2. Drug Preparation: IBP Inhibitors

Fluvastatin was purchased from Selleck Chemicals (Houston, TX, USA; Cat# S1909) and suspended in DMSO at a stock concentration of 10 mM. Digeranyl bisphosphonate (DGBP), a GGDPS inhibitor, was acquired under an MTA from Terpenoid Therapeutics Inc. by Penn State College of Medicine [13].

Farnesyl transferase inhibitor (FTI-277) was purchased from Sigma-Aldrich (St. Louis, MO, USA; Cat# F9803), and geranylgeranyl transferase inhibitor (GGTI-286) was purchased from Selleck Chemicals (Houston, TX, USA; Cat# S7466). Compounds were suspended in DMSO at a stock concentration of 100 mM. Drug dose–response experiments were performed in triplicate using MTT assays to determine suitable concentrations for all experiments.

### 2.3. Addbacks

To assess the rescue capabilities of IBP intermediates after statin treatment, addbacks of the IBP activators of mevalonic acid (MVA; 250 μM), FOH (20 μM), and GGOH (30 μM) were added to fluvastatin-treated wells/plates after a 24 h period. They were then left for another 24 h prior to stopping the experiment. FPP and GGPP were added back as their alcohol forms (farnesol (FOH) and geranylgeraniol (GGOH)) due to the original metabolites’ impermeability to the cell membrane and instability. MVA was purchased from Sigma-Aldrich (St. Louis, MO, USA; Cat# 90469). FOH and GGOH were obtained from (Thermo Fisher Scientific, Waltham, MA, USA).

### 2.4. Cell Treatment

VN-MCC13 cells were plated in 12-well plates at a seeding density of 1 × 10^5^ and incubated for 24 h prior to treatment, allowing cells to reach approximately 70% confluency. Due to their nature as suspension cells, VP-MKL-1 cells were plated and incubated for 3 h prior to treatment. Unless otherwise stated, all cells were treated for 48 h. Cells were treated with fluvastatin alone, fluvastatin plus individual addback conditions (as described above), and with downstream IBP inhibitors (FTI-277, GGTI-286, or DGBP). FTI-277 and GGTI-286 were used to target farnesyl transferase and geranylgeranyl transferase, respectively, with the intended inhibition of prenylation of Ras (farnesylated) and Rho (geranylgeranylated) GTPases. DGBP was employed to target geranylgeranyl diphosphate synthase (GGDPS), thereby depleting GGPP.

### 2.5. Cell Viability Assays

Crystal violet assays were initially performed to assess cellular viability of VN-MCC13 cells in response to addbacks of MVA, FOH, and GGOH following fluvastatin treatment. Cells were plated in 12-well plates at a seeding density of 1 × 10^5^. VN-MCC13 cells were treated with a range of fluvastatin doses (3 μM, 10 μM, 30 μM) for a total of 48 h. Addbacks of MVA (250 μM), FOH (20 μM), and GGOH (30 μM) were administered after 24 h of treatment. Cells were fixed with ice-cold methanol for 10 min and then then stained with crystal violet for 20 min. Plates were read on the SpectraMax i3x reader at 570 nm, and results were calculated as a percentage of the control.

Following IBP perturbation, cellular metabolic activity was measured using the MTT assay. VN-MCC13 and VP-MKL-1 cells were plated in 96-well plates at seeding densities of 9000 and 30,000 cells/well, respectively. Cells were treated for 48 h with IBP inhibitors (fluvastatin, DGBP, FTI, or GGTI) using a range of doses (0.01 μM–100 μM) to establish viability curves. After 24 h of fluvastatin treatment, cells received addbacks of MVA (250 μM), FOH (30 μM), and GGOH (20 μM) for a further 24 h. At 44 h, 10 μL of MTT solution (5 mg/mL) was added, and at 48 h, stop solution (80% isopropanol, 10% Triton X-100, 10% 1 N HCl) was added. Plates were incubated overnight at 37 °C and then read on a SpectraMax i3x reader at 570 nm (test) and 690 nm (background). Results were calculated by subtracting the wavelength from the background values and were then represented as a percentage of the control.

### 2.6. Flow Cytometry

VN-MCC13 cells (90,000 cells/well) and VP-MKL-1 cells (200,000 cells/well) were plated in 12-well plates and treated with IBP inhibitors (fluvastatin, FTI, GGTI, or DGBP) for 48 h. Cells were collected and washed in FACS buffer (DPBS without calcium and magnesium, 1% FBS, 2 mM EDTA). Etoposide (20 μM) was employed as a positive control throughout the experiments, as it is widely accepted as an apoptotic agent [14]. For Annexin V/PI staining, cells were resuspended in Annexin V binding buffer (10 mM Hepes, 140 mM NaCl, 2.5 mM CaCl_2_, water) and stained with the APC Annexin V Apoptosis Detection Kit (BioLegend, San Diego, CA, USA; Cat# 640932) for 15 min before being analyzed. For the Annexin V/Live-Dead staining, cells were resuspended with the LIVE/DEAD™ Fixable Near-IR Dead Cell Stain Kit (Invitrogen™, Waltham, MA, USA; Cat# L10119) for 30 min at 4 °C and then permeabilized and fixed using the eBioscience™ Foxp3/Transcription Factor Staining Buffer Set (Invitrogen™, Waltham, MA, USA; Cat# 00-5523-00). To inhibit RNA binding, cells were treated with RNase I (Thermo Scientific™, Waltham, MA, USA; Cat# EN0602) and then resuspended in PI stain for 15 min. All final cell suspensions contained known concentrations of CountBright^TM^ Absolute Counting Beads (Thermo Scientific^TM^, Waltham, MA, USA, Cat# C36950). The ratio of known bead input to bead output was calculated to determine absolute cell counts in each sample. Samples were analyzed using the BD Biosciences BD FACSymphony™ 17 and 23 cytometers at the Penn State College of Medicine Flow Core Facility. Analysis was performed using the FlowJo 10.8 data analysis software. Gating strategies for MCC13 and MKL-1 are presented in Supplemental Figures S2 and S3 accordingly.

### 2.7. Quantitative Real-Time PCR (RT-qPCR)

Following the treatment conditions described above, cells were collected, and RNA was extracted using the Qiagen RNeasy Mini Kit (Hilden, Germany, Cat# 74104). cDNA synthesis was carried out using SuperScript™ III Reverse Transcriptase (Invitrogen, Waltham, MA, USA, Cat# 18080093) following the manufacturer’s instructions. Samples were then combined with TaqMan primers and were plated in 96-well plates. RT-qPCR was performed using a Bio-Rad CFX96 Real-Time PCR Detection System. HMGCR was employed as a positive control, and GAPDH was employed as the internal control. Relative gene expression levels were calculated using the 2^−ΔΔCt^ analysis method [15] and represented as fold changes in mRNA relative to control samples.

The following TaqMan primers were used: GAPDH (Hs02786624_g1), HMGCR (Hs00168352_m1), HRAS (Hs00978050_g1), NRAS (Hs00180035_m1), KRAS (Hs00364284_g1), RHOA (Hs00357608_m1), CDC42 (Hs00918044_g1), and RAC1 (Hs01902432_s1).

### 2.8. Immunoblotting

Following the treatment conditions described above, cells were collected and lysed using cell lysis buffer (Part # CLB01) supplemented with a protease inhibitor cocktail (Cat# PIC02). To control for equal protein loading, protein concentrations were determined using a Pierce™ bicinchoninic acid (BCA) assay (Thermo Fisher Scientific, Waltham, MA, USA), following the manufacturer’s instructions. For separation, 20 μg of protein was loaded onto 12% SDS-polyacrylamide gels and then transferred to PVDF membranes. Membranes were blocked with blocking buffer (Cat# R8207) and probed with primary and secondary antibodies. Dried membranes were read using the ScanLater™ Western Blot (Molecular Devices, San Jose, CA, USA) attachment on the SpectraMax i3x reader. Blots were stripped using Pierce™ Restore™ PLUS Western Blot Stripping Buffer (Cat# 46428, Thermo Fisher Scientific) for five minutes and then washed with ScanLater wash buffer and reprobed with primary and secondary antibodies, as described below.

The following primary antibodies were used at a dilution of 1:1000: anti-RhoA (2117s), anti-Cdc42 (ab187643), anti-Rac1/2/3 (ab282581), and anti-vinculin (13901S). ScanLater Europium-conjugated anti-mouse or anti-rabbit secondary antibodies (Molecular Devices, San Jose, CA, USA; Cat# R8205, Cat# R8209) were utilized at a dilution of 1:5000.

Unmodified western blot images can be found in the supplemental section (Figures S4–S6).

### 2.9. Pull-Down GST Assay

After cell treatment with IBP inhibitors (fluvastatin, FTI-277, GGTI-286, or DGBP), VP-MKL-1 cells were collected and lysed as described previously. Pull-down experiments were carried out using the RhoA and Cdc42/Rac1 Pull-Down Activation Assay Biochem Kits™ (Cytoskeleton, Inc., Denver, CO, USA; Cat# BK036 and Cat# BK035). Samples to be measured for active GTP-bound protein were first incubated at 4 °C for 1 h with either rhotekin-RBD (RhoA) or PAK-RBD (Rac1/Cdc42) beads and then centrifuged and washed with wash buffer. Protein content was quantified via immunoblotting using protocols previously described for immunoblotting experiments. GTP-bound samples were compared against positive (GTPγS) and negative (GDP) protein controls. Non-pulled-down total protein samples (20 μg) were loaded alongside GTP samples; however, these were not compared quantitatively to one another, as the protein amounts loaded were substantially differently.

### 2.10. Statistical Analysis

The results are representative of three independent biological experiments, with two or more technical replicates where possible. One-way ANOVA with Dunnett’s multiple-comparison test was used when comparing a single variable across groups, and two-way ANOVA was utilized with Šídák’s multiple-comparison test when two or more variables were compared. Data points are represented as mean values ± SEM, and statistical significance is defined as * *p* < 0.05, ** *p* < 0.01, *** *p* < 0.001, and **** *p* < 0.0001. Data analysis and figure generation were performed using GraphPad Prism software (version 11.0.0).

## 3. Limitations and Future Directions

This work has several limitations. First, experiments were performed at a fixed timepoint of 48 h, in line with previous research using similar compounds and cell lines [16,17]. While prenylation is a stable, irreversible modification, the 48 h timepoint may not capture earlier or more dynamic changes in cellular responses. Future studies will build on these findings by incorporating multiple timepoints to provide a more comprehensive understanding of the temporal effects of IBP inhibition. Additionally, although we did not directly test whether FOH and GGOH were converted to their prenylated counterparts when added to cells, exogenous FOH and GGOH are widely used by our group and others to rescue prenylation [10]. Prior research has suggested that a ‘novel salvage pathway’ utilizes an endogenous enzymatic process for the phosphorylation of FOH and GGOH to restore FPP and GGPP [18,19].

To measure cellular metabolism and viability, we focused on MTT assays and flow cytometry. Although we also utilized crystal violet staining in the VN-MCC13 line as an additional measure of viability, we could not extend this experiment to VP-MKL-1 cells due to their suspension-growth properties. Crystal violet assays are typically only compatible with adherent cell lines. Future studies could incorporate additional measures of viability to confirm and extend our findings. In particular, treatment concentrations were determined via MTT assay, which is limited to measuring drug effects on metabolic activity. While MTT assays are widely used to infer cellular viability directly from metabolic activity readouts, these variables are not always agreeable; thus, secondary viability methods are necessary to confirm findings [20].

Furthermore, our analysis of RAS activity focused on mRNA expression, as although we attempted to complement mRNA and protein assays, Ras protein content was consistently low and challenging to detect. This was not surprising given the low mRNA expression measured previously; however, future studies utilizing more sensitive protein detection methods should be conducted to strengthen the link between transcriptional changes and downstream protein expression. Unexpectedly, Rho protein expression increased following IBP inhibition. Although we performed GTPase pull-down assays to understand this paradoxical increase, our interpretation is limited by a lack of knowledge regarding subcellular localization, and we aim to investigate this in future work. MKL-1 cells exhibited a high baseline level of apoptosis, which may confound interpretation of treatment-induced effects. This could explain why rescue experiments with IBP intermediates did not elicit strong responses in VP cells compared to VN cells, since the high levels of apoptosis may have prevented restoration of growth beyond a certain capacity. Future work should utilize VP cell lines that exhibit lower baseline apoptosis levels to better interpret treatment-specific effects.

While statins and other IBP inhibitors resulted in growth inhibition, the concentrations required to achieve these effects were sometimes as high as 100 μM, which, based on prior studies, is not a therapeutically realistic dose. Due to the increased complexity of cellular interactions in a three-dimensional model, spheroids and other in vivo models may be more sensitive to lower drug concentrations; however, the use of high treatment concentrations in cell-based studies is not uncommon, increasing the challenge of translating such work into a clinical setting [16,21,22].

Finally, experiments were carried out exclusively in cell lines, which was appropriate for initial drug screening and exploration of the research questions; however, in vitro models have inherent limitations in their applicability. The growth conditions of two-dimensional cell culture may affect cellular properties such as metabolism, proliferation, and responsiveness to drugs, thereby limiting the ability of the results to capture the complexity of systems involving cell–cell interaction. To improve cell–cell signaling, the next step in this work would be to utilize three-dimensional spheroid models to expand upon our findings, as our group has developed a successful spheroid model.

## 4. Results

### 4.1. Inhibition of the IBP Induces Dose-Dependent Cytotoxicity in MCC Cells

To investigate the importance of the IBP for cellular viability in MCC cell lines, VN-MCC13 cell were initially treated with fluvastatin and simvastatin. A range of concentrations (0.3 μM–30 μM) was used to establish dose–response curves, and cellular metabolic activity was assessed using an MTT assay. Dose-dependent decreases in cellular metabolic activity were observed following statin treatment, with the strongest effects observed following fluvastatin treatment (Figure 1B). Based on these results, fluvastatin was used in subsequent experiments.

**Figure 2:**
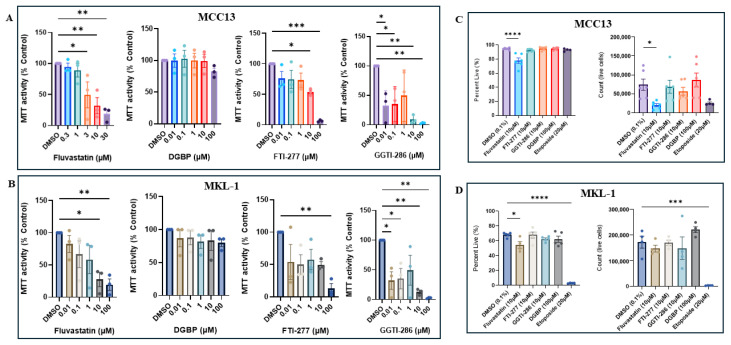
Treatment with IBP inhibitors decreases cellular viability across viral-negative and viral-positive MCC lines. Effects of IBP inhibitors (fluvastatin, DGBP, FTI-277, and GGTI-286) on metabolic activity in (**A**) MCC13 cells and (**B**) MKL-1 cells. Live/dead flow cytometry analysis of (**C**) MCC13 cells and (**D**) MKL-1 cells following treatment with IBP inhibitors. All MCC13 and MKL-1 data are represented in their own separate color palettes. Data points represent independent experiments (n = 3), with three technical replicates per experiment. Error bars indicate mean values ± SEM. * *p* < 0.05, ** *p* < 0.01, *** *p* < 0.001, and **** *p* < 0.0001.

To determine whether downstream intermediates of the IBP similarly affected metabolic activity, VN-MCC13 and VP-MKL-1 cells were treated with the downstream pathway inhibitors DGBP, FTI-277, or GGTI-286 across a range of doses (0.01 μM–100 μM) and analyzed using an MTT assay. Following DGBP treatment, we observed modest decreases in cellular metabolic activity at higher concentrations. In contrast, following FTI-277 and GGTI-286 treatment, we observed strong concentration-dependent reductions in cellular metabolic activity (Figure 2A,B). Among the four compounds tested, DGBP produced the weakest reduction in metabolic activity. Across the four compounds tested, both cell lines exhibited similar decreases in metabolic activity.

To further assess cellular behavior following IBP perturbation, cells were treated with IBP inhibitors, fixed with a fixable live/dead stain, and then analyzed by flow cytometry. Among the five compounds tested, we only observed significant decreases in viability across lines following statin treatment, whereas the other compounds did not significantly alter viability or absolute cell counts (Figure 2C,D). Across cell lines, GGTI-286-treated cells displayed decreases in absolute cell counts, while DGBP-treated cells displayed increased absolute cell counts. Etoposide, an internal control for apoptosis studies, exhibited a particularly potent effect on the VP-MKL-1 line, in which we observed a dramatic reduction in viability and absolute cell counts (Figure 2D).

### 4.2. Fluvastatin Differentially Affects Cell-Cycle Dynamics in Viral-Negative and Viral-Positive MCC Lines

**Figure 3:**
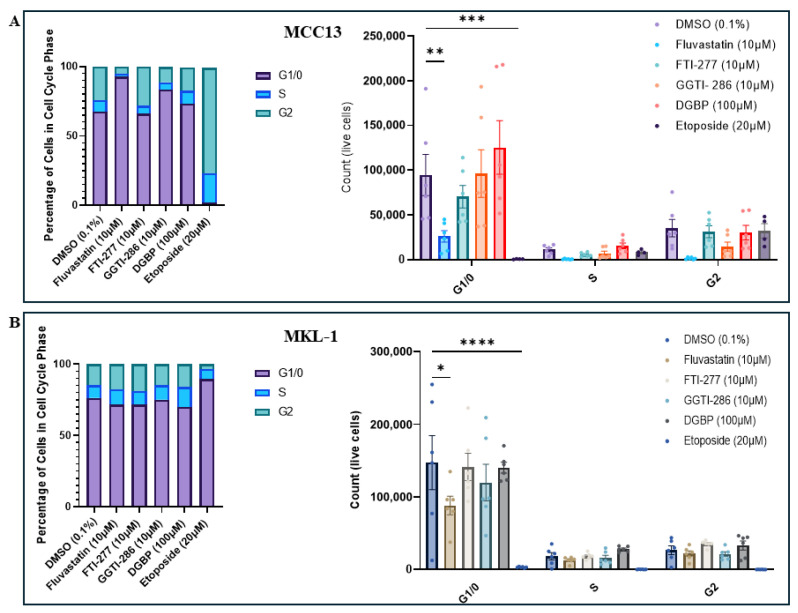
Cell-cycle phase distribution is differentially affected by IBP modulation across viral-negative and viral-positive MCC cell lines. Percentages of viable cells and absolute cell counts across cell-cycle phases (G0/1, S, and G2) in (**A**) MCC13 cells and (**B**) MKL-1 cells, following treatment with IBP inhibitors. All MCC13 and MKL-1 data are represented in their own separate color palettes. Data points represent independent experiments (n = 3), with three technical replicates per experiment. Error bars indicate mean values ± SEM. * *p* < 0.05, ** *p* < 0.01, *** *p* < 0.001, and **** *p* < 0.0001.

Cell lines were permeabilized and stained with propidium iodide (PI) to assess DNA content, and cell-cycle distribution was analyzed by flow cytometry following IBP inhibitor treatment. In the VN-MCC13 line, fluvastatin and GGTI-286 treatment increased the proportion of cells in the G0/1 phase relative to controls, whereas FTI-277 and DGBP treatment did not significantly alter cell-cycle distribution (Figure 3A). In contrast, in the VP-MKL-1 line, no treatment altered the proportion of cells in the G0/1 phase or otherwise affected cell-cycle phase distribution. Similar to the observations in VN-MCC13 cells, statin treatment led to decreases in the number of viable cells in the G0/1 phase in VP-MKL-1 cells (Figure 3B). Overall, both lines exhibited a high baseline proportion of cells residing in the G0/1 phase.

### 4.3. IBP Inhibitors Differentially Affect Apoptosis in Viral-Negative and Viral-Positive MCC Lines

**Figure 4:**
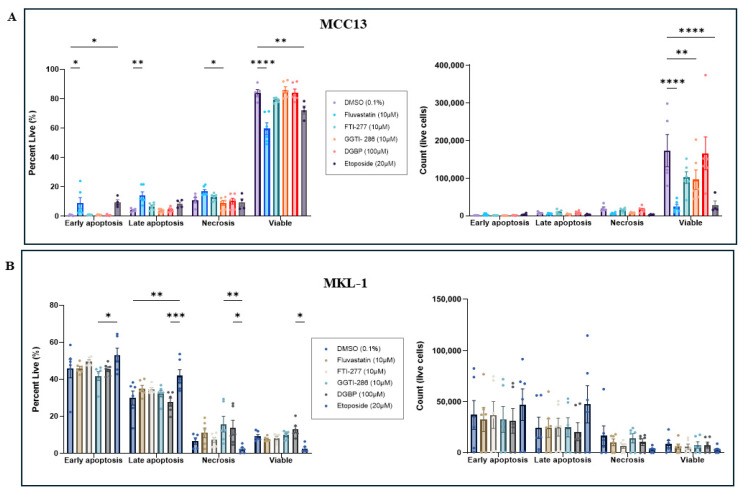
Statins induce apoptosis in MCC13 cells but not MKL-1 cells. (**A**) MCC13 cells and (**B**) MKL-1 cells were assessed for percentages of viable cells and absolute viable cell counts following treatment with IBP inhibitors. Viability, necrosis, and early/late apoptosis were measured using flow cytometry. All MCC13 and MKL-1 data are represented in their own separate color palettes. Data points represent independent experiments (n = 3), with three technical replicates per experiment. Error bars indicate mean values ± SEM. * *p* < 0.05, ** *p* < 0.01, *** *p* < 0.001, and **** *p* < 0.0001.

To evaluate the apoptotic profiles of MCC cell lines following IBP perturbation, cells underwent Annexin V and PI staining and were analyzed using flow cytometry. Again, a large discrepancy was observed between the two cell lines regardless of treatment, with VN-MCC13 cells exhibiting high viability, whereas VP-MKL-1 cells contained a higher proportion of cells undergoing early or late apoptosis. In the VN-MCC13 line, we observed a significantly increased percentage of cells undergoing early and late apoptosis, along with a decrease in viable cell numbers following fluvastatin treatment (Figure 4A). Following FTI-277 and GGTI-286 treatment, cells exhibited decreases in viable cell counts. In the VP-MKL-1 line, IBP inhibitors did not significantly alter the apoptotic profile of the cells (Figure 4B).

### 4.4. GGPP Restores Cellular Viability Across Viral-Negative and Viral-Positive Cell Lines

**Figure 5:**
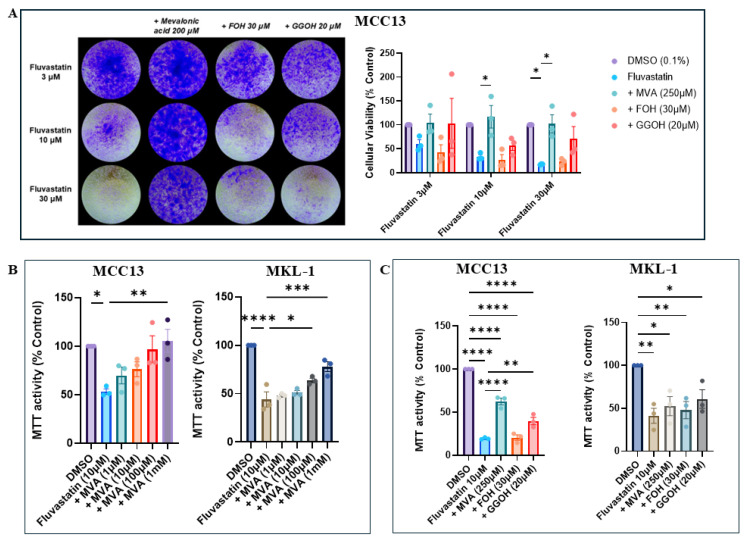
GGPP rescues growth in statin-treated MCC cell lines. (**A**) MCC13 cells were assessed for their response to MVA, FOH, and GGOH addbacks following fluvastatin treatment (3 μM, 10 μM, and 30 μM). Cells were stained with crystal violet, and representative images are shown. (**B**) MTT assays were used to assess the cellular viability of MCC13 and MKL-1 cells in response to MVA titration following a fixed fluvastatin treatment (10 μM). (**C**) MCC13 and MKL-1 cells were treated with fluvastatin (10 μM), followed by addbacks of MVA, FOH, and GGOH. Viability was measured using MTT assays. All MCC13 and MKL-1 data are represented in their own separate color palettes. Data points represent independent experiments (n = 3), with each experiment performed with three technical replicates. Error bars indicate mean values ± SEM. * *p* < 0.05, ** *p* < 0.01, *** *p* < 0.001, and **** *p* < 0.0001.

To determine whether the observed loss of cellular viability was due to depletion of the specific downstream isoprenoids FPP or GGPP, VN-MCC13 cells were treated with increasing concentrations of fluvastatin for 24 h (3 μM, 10 μM, and 30 μM), followed by addbacks of the intermediates MVA, FOH, or GGOH for a further 24 h. Crystal violet staining was utilized to measure cellular viability, and MVA was utilized as a positive control to ensure that the observed effects were due to targeting of the IBP. Across concentrations, addbacks of GGOH, but not FOH, rescued cell growth to control levels, suggesting a preferential reliance on geranylgeranylation for cellular viability (Figure 5A).

To further investigate cellular viability, VN-MCC13 and VP-MKL-1 cells were treated with a single dose of fluvastatin (10 μM) in an MTT assay format, with intermediate addbacks administered after 24 h (Figure 5C). Consistent with the crystal violet results, VN-MCC13 cells responded robustly to GGOH addbacks, but not FOH addbacks, whereas VP-MKL-1 cells exhibited moderate rescue with GGOH. Notably, GGOH partially restored viability to levels similar to or exceeding those achieved with MVA. Dose–response experiments confirmed that MVA could rescue cellular viability when applied at sufficiently high concentrations (Figure 5B). To ensure that effects were not due to FOH or GGOH related toxicity, we supplemented untreated cells with FOH and GGOH for 24 h and found no significant changes in MTT activity (Figure S1).

### 4.5. Analysis of Ras and Rho Family mRNA Expression Following IBP Perturbation

To investigate whether changes in cellular viability were due to fluctuations in Ras and Rho signaling, three *Ras* (*HRAS*, *NRAS*, and *KRAS*) and *Rho* (*RHOA*, *CDC42*, and *RAC1*) family genes were measured using RT-qPCR. Each cell line received addbacks of MVA, FOH, or GGOH following fluvastatin treatment, in addition to treatment with IBP inhibitors (FTI, GGTI, or DGBP).

**Figure 6:**
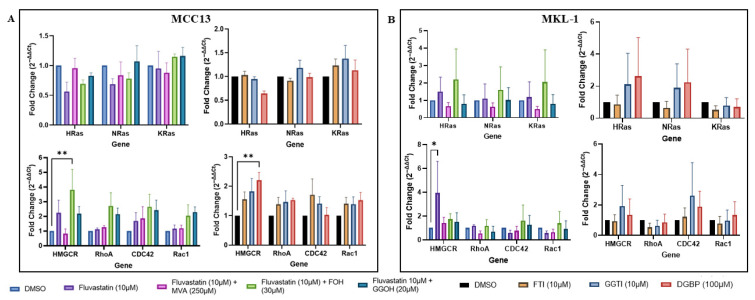
*RAS* and *RHO* mRNA levels are differentially perturbed in MCC13 and MKL-1 cell lines after IBP inhibition (**A**) MCC13 and (**B**) MKL-1 cells were analyzed for their relative mRNA levels of *RAS* and *RHO* GTPases following treatment with fluvastatin alone, fluvastatin plus IBP addbacks (MVA, FOH, and GGOH), and downstream IBP inhibitors (FTI, GGTI, and DGBP). Gene expression was quantified via qPCR and normalized to both a housekeeping gene *(GAPDH)* and DMSO controls. Data points represent independent experiments (n = 3), with each experiment performed with three technical replicates. Error bars indicate mean values ± SEM. * *p* < 0.05 and ** *p* < 0.01.

In the VN-MCC13 line, *Ras* mRNA expression was not significantly altered by any treatment; however, trends indicated that statin-treated cells exhibited decreased *HRAS* and *NRAS* expression, and addback treatment restored expression toward control levels. *RHO* mRNA expression was not sensitive to statin treatment; however, increases were observed with FOH and GGOH supplementation across *Rho* genes. Cells treated with IBP inhibitors also exhibited increased *Rho* mRNA expression, and notably, DGBP treatment significantly increased *HMGCR* expression (Figure 6A).

In the VP-MKL-1 line, GGTI-286 and DGBP treatment increased *HRAS* and *NRAS* mRNA expression. Following fluvastatin treatment, addbacks of MVA and GGOH, but not FOH, restored *HRAS* levels to control levels. Similar to VN-MCC13 cells, in VP-MKL-1 cells, fluvastatin treatment did not alter *Rho* mRNA expression; however, trends toward increased *CDC42* mRNA expression were observed following GGTI-286 and DGBP treatment (Figure 6B).

### 4.6. Perturbation of the IBP Induces Variable Changes in Rho GTPase Protein Expression

**Figure 7:**
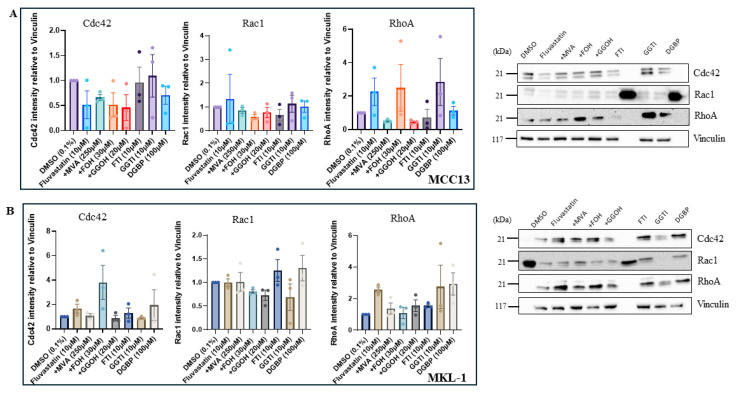
Rho GTPase expression varies following treatment with IBP inhibitors. (**A**) Cdc42, Rac1, and RhoA protein levels were measured in MCC13 cells and (**B**) MKL-1 cells following treatment with fluvastatin alone, fluvastatin plus isoprenoid addbacks (MVA, FOH, and GGOH), and downstream IBP inhibitors (FTI-277, GGTI-286, and DGBP). Representative immunoblotting images are shown. All MCC13 and MKL-1 data are represented in their own separate color palettes. Data points represent independent experiments (n = 3), with bars indicating mean values ± SEM.

Western blot analysis was performed across cell lines to determine whether the transcriptional changes observed were consistent with protein expression levels. Across both cell lines, no statistically significant differences were observed following treatment; however, several trends were identified. Although the effects of fluvastatin treatment on Rho protein expression were variable in both lines, protein expression was typically restored to control levels following MVA supplementation. In some cases, GGOH supplementation produced a similar effect. FOH addbacks increased RhoA expression in VN-MCC13 cells (Figure 7A) and Cdc42 expression in VP-MKL-1 cells (Figure 7B). Unexpectedly, fluvastatin-treated cells exhibited a twofold increase in RhoA expression and, in VP-MKL-1 cells only, Cdc42 expression. Additionally, RhoA expression increased following DGBP treatment in VP-MKL-1 cells and following GGTI-286 treatment across both cell lines. Overall, Rac1 expression appeared unchanged.

### 4.7. IBP Perturbation Affects the Activation State of Rho GTPase Proteins in VP-MKL-1 Cells

**Figure 8:**
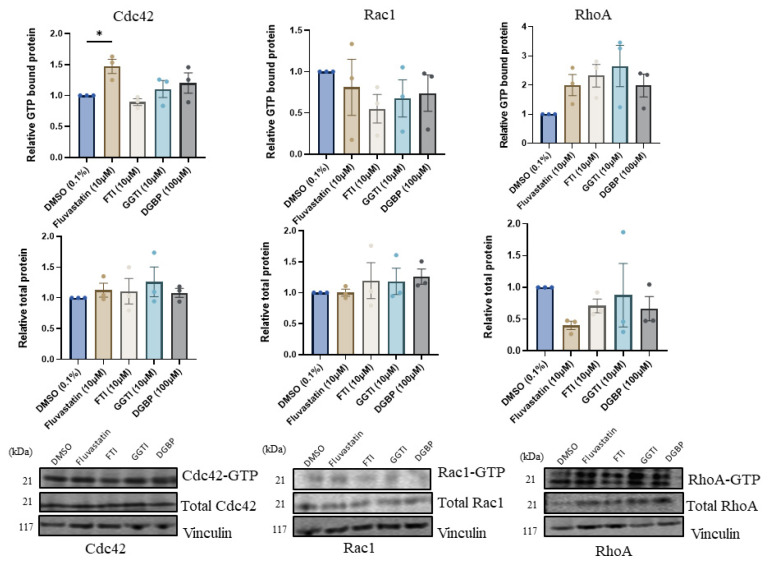
Fluvastatin increases active Cdc42 protein expression in MKL-1 cells. Cdc42, Rac1, and RhoA GTP-bound (active) protein levels were measured in MKL-1 cells following treatment with IBP inhibitors (fluvastatin, FTI-277, GGTI-286, and DGBP). Active GTP-bound proteins in samples were collected using a GTP pull-down assay, after which protein levels were measured via Western blotting. Relative total protein levels are shown for comparison. Data points represent independent experiments (n = 3), with three technical replicates per experiment. Error bars indicate mean values ± SEM. * *p* < 0.05.

Due to the transcriptional and protein expression changes observed in the previous experiments, VP-MKL-1 cells were assessed to determine how IBP perturbation affected the activation state of Rho proteins. Following treatment with IBP inhibitors (fluvastatin, FTI, GGTI, or DGBP), fluvastatin treatment significantly increased GTP-bound Cdc42 expression relative to controls (Figure 8). Although not statistically significant, cells exposed to IBP inhibitors displayed about a twofold increase in active RhoA expression, with the most prominent increase observed following GGTI treatment.

## 5. Discussion

In this study, we investigated the effects of inhibition of the isoprenoid biosynthesis pathway in Merkel cell carcinoma cell lines with the goal of expanding the knowledge base on a previously unexplored topic. Using specific compounds designed to inhibit enzymes throughout the pathway, we were able to narrow down the key isoprenoid intermediates involved in pro-tumorigenic phenotypes in MCC cell lines. Through an active comparison of VN-MCC13 and VP-MKL-1 MCC cell lines, we identified differences in their cellular responses to IBP inhibition and dependence on downstream prenylation.

Across MCC cell lines, we observed perturbation of metabolic activity in a concentration-dependent manner following treatment with IBP inhibitors, namely fluvastatin, FTI-277, and GGTI-286. A less pronounced decrease in metabolic activity was observed with the GGDPS inhibitor DGBP than with fluvastatin, indicating that specific intermediates of the pathway, such as mevalonic acid, could be more critical for metabolic processes than others, such as GGPP. Additionally, cells treated with prenyl transferase inhibitors exhibited similarly pronounced decreases in cellular metabolism, suggesting a reliance on small GTPases for cellular survival. In combination with the MTT results, live/dead staining revealed distinct phenotypic responses to IBP perturbation. While statin treatment remained the most effective inhibitor, decreasing both viability and absolute cell counts, the other IBP inhibitors did not exhibit the same trends. For FTI-277 and GGTI-286, the decreases in metabolic activity observed with the MTT assay were either mild or absent when measured using live/dead staining. In line with other reports, these findings suggest that downstream inhibition of the IBP leads to suppression of metabolic activity but does not completely kill MCC cells. In other cancers, FTI and GGTI have been shown to inhibit cell growth through cytostatic mechanisms, such as disrupting cell-cycle dynamics or induction of apoptosis [23,24,25]; however, drug effects vary across cancer types, highlighting the cancer-specific variability in dependence on prenylation.

To further analyze the discrepancy observed between metabolic activity and viability, we analyzed cell populations for changes in cell-cycle dynamics and found that in VN-MCC13 cells, fluvastatin- and GGTI-289-treated cells exhibited G1/G0 arrest compared to controls, consistent with reports in other cancer subtypes [26,27]. This finding suggests that inhibition of mevalonic acid and geranylgeranylation can increase cellular senescence, driving VN-MCC13 cells into a cytostatic state [28]. In contrast, treatment did not significantly alter VP-MKL-1 cells’ cell-cycle phase distribution, perhaps because these cells exist at a high baseline proportion in G0/1 and thus IBP manipulation may not push cells into further arrest. When apoptosis and necrosis levels were measured, statin-treated cells exhibited increased percentages and counts of VN-MCC13 cells undergoing early and late apoptosis, while FTI-277 and GGTI-286 treatment decreased the total number of viable cells, further corroborating that prenylation inhibition in VN-MCC13 cells increases cell death. In comparison, VP-MKL-1 cells exhibited high baseline proportions of cells undergoing early apoptosis, late apoptosis, and necrosis, with IBP perturbation not affecting these percentages. Again, the high baseline apoptosis observed in this cell line could explain why these cells are less sensitive to IBP perturbation with respect to cellular survival; however, it does not explain the changes in cellular metabolism measured previously. While our results show that etoposide functions successfully as a positive control, it should be noted that others have found that healthy VP-MKL-1 cells exhibit constitutive binding to Annexin V; thus, these results may be limiting [8].

To better narrow down the isoprenoid intermediates that could drive the effects observed in previous assays, cells were subjected to addbacks of the intermediates MVA, FOH, or GGOH following fluvastatin treatment. FOH and GGOH were used as alternatives to FPP and GGPP, as these alcohols readily enter membranes and are converted to their prenyl counterparts once inside the cell, although the exact enzymes responsible for this process remain undetermined [29]. In VN-MCC13 cells, GGOH, but not FOH, restored cellular viability to control levels. While GGOH rescue was not as pronounced in VP-MKL-1 cells, the rescue effect matched that observed with MVA addbacks and suggests that geranylgeranylation may contribute to the observed loss of cellular viability across cell lines.

Due to the suspected involvement of prenylation in cellular survival in MCC, key prenylated proteins, Ras and Rho GTPases, were evaluated for mRNA expression following IBP manipulation. This focus is supported by other reports identifying gene mutations in PIK3CA and HRAS in MCC, along with suspected involvement of the RTK-RAS pathway, together suggesting that the metabolic pathways driving prenylation could support tumorigenic processes in MCC [30,31]. Overall, RAS mRNA expression was not significantly altered by treatment, indicating that farnesylation may not explain the changes in cellular phenotypes observed previously. Across cell lines and particularly in VP-MKL-1 cells, trends were observed in which IBP inhibitors increased Rho mRNA expression. Notably, inhibition of GGPP with DGBP in VP-MKL-1 cells produced a twofold increase in HMGCR mRNA expression, matching the increase induced by statins. HMGCoA is considered the key intermediate in the IBP, with HMGCR acting to maintain tight control of the pathway [32]; thus, a significant increase in its expression following DGBP treatment points to a role for GGPP in regulation of the IBP.

Protein analysis of Rho GTPases was utilized to investigate the observed increases in Rho mRNA expression following IBP inhibition and to better understand the role of these geranylgeranylated proteins. Based on a previous observation that Cdc42 and RhoA were key drivers of cellular motility in VP-MCC cells [33], we expected our treatments to significantly decrease expression of these proteins. Contrary to our expectations, protein expression was variable, with the most notable trend being increased RhoA expression across cell lines following fluvastatin and GGTI-286 treatment. Further analysis in the VP-MKL-1 line showed increases in active GTP-bound Cdc42 following fluvastatin treatment and in GTP-bound Rho following inhibitor treatments. Because statins have been reported to consistently reduce Rho expression, we hypothesize that this paradoxical increase in GTP loading could reflect an increase in total Rho protein levels, with the unprenylated form accounting for most of this increase [34].

Emerging evidence suggests that Rho GTPases can exist in active GTP-bound states outside the plasma membrane, including in the cytosol or within nuclear compartments, where their cellular function is altered [35,36]. As we did not investigate the localization of these proteins, it is impossible to comment on whether the observed GTP-bound pools are membrane-associated. It is possible that the observed accumulation of GTP-bound RhoA and Cdc42 could therefore represent mislocalized GTPase pools in a non-canonically active state that are unable to interact with downstream effectors, potentially contributing to the observed changes in cellular viability. To investigate whether the observed accumulation is representative of prenylated or unprenylated protein, fractionation studies could be utilized in the future to measure membrane vs. cytosolic protein distribution. Prenylated forms would be membrane-bound, whereas unprenylated forms would be found in the cytosol, thereby confirming whether the observed increases are functional.

We determined that perturbation of the IBP differentially impacts cellular phenotypes in VN-MCC and VP-MCC cell lines and that in VN-VN-MCC13 cells, these effects can be attributed to loss of geranylgeranylation; thus, a cytostatic phenotype ensues in which cells undergo G0/1 arrest. These findings indicate that statins could represent a potential means of reducing neoplasia in viral-negative cases; however, to ensure more potent effects, synergistic treatment strategies should be investigated. Of note, etoposide’s pronounced cytotoxic effect in VP-MKL-1 cells suggests that these cells are susceptible to apoptosis when DNA damage is induced. Of the treatments tested, etoposide was the only agent to both increase apoptosis and decrease viability. In line with other work, in VP-MCC cells, decreased proliferative capacity has been linked to a p53-dependent apoptotic cell death response [37]. These findings highlight VP-MKL-1 cells’ reliance on intact DNA damage repair mechanisms for survival and point to a potential vulnerability of VP-MCC cells that should be explored further.

A possible explanation for the differences in statin sensitivity observed between cell lines could, in part, be due to their mesenchymal traits, as some researchers have shown that cell lines that are more sensitive to statins are characterized as mesenchymal-like [38]. There is evidence showing that VN-MCC cells fall within the mesenchymal-like category due to their high expression of epithelial–mesenchymal transition (EMT)-related genes [39], whereas VP-MCC cells exhibit more of a neuroendocrine profile, with lower expression of EMT-related markers [40]. Importantly, this difference in cellular state may reflect varying signaling dependencies on the IBP [41]. Cells with mesenchymal characteristics often exhibit enhanced cytoskeletal remodeling and motility signaling, which rely on prenylated GTPases such as Rho proteins to promote migratory processes [42,43]. It is possible that VN-MCC cells display increased sensitivity to disruption of IBP-dependent processes, whereas VP-MCC cells may be less directly dependent on IBP processes and instead exhibit a neuroendocrine transcriptional profile with low expression of EMT-associated genes. As a result, VP-MKL-1 cells may exhibit reduced dependence on the IBP for cellular growth and survival; however, as IBP inhibitors alter GTPase expression in these cells, Rho GTPases may still have functional importance. It is likely that alternative downstream signaling pathways, such as MAPK/ERK or PI3K/AKT [44,45], could explain the GTPase changes observed following IBP inhibition.

## 6. Conclusions

While a handful of studies have claimed that statin use is positively correlated with an increased risk of poor MCC prognosis [46,47,48], no studies have yet explored the in vitro impact of statin treatment or the involvement of the IBP in MCC tumorigenesis. In this study, we investigated whether inhibition of the IBP can reduce pro-carcinogenic phenotypes such as proliferation and altered cellular dynamics in MCC. We determined that IBP inhibition perturbs metabolic activity and viability in MCC cell lines and that GGPP is important for cellular survival. Additionally, our results suggest that VP-MCC and VN-MCC cell lines exhibit differential reliance on the IBP, as VN-MCC cells were more sensitive to IBP inhibition, exhibiting cellular changes such as cell-cycle arrest and increased apoptosis in response to treatment. Our results highlight differences between VPMCC and VN-MCC cell lines, which may indicate that MCPyV alters cellular responses, which could potentially translate to the clinical setting; however, in vivo studies are required to further explore IBP metabolism in MCC. While similarities between VP-MCC and VN-MCC molecular signatures have been reported, differential molecular dependencies and uncertain pathophysiological mechanisms between the two subtypes present a gap in knowledge of MCC that should be investigated further [2]. Much current research focuses on MCPyV genome integration and the role it may play in dictating molecular mechanisms; thus, future work would benefit from further elaborating on the interplay between MCPyV and cellular metabolism.

## Data Availability

All data generated in this work are included in the figures of the manuscript. The data supporting the findings are stored at the Penn State College of Medicine in the laboratory of the corresponding author and are available upon request.

## Supplementary Materials

The following supporting information can be downloaded at https://www.mdpi.com/article/10.3390/cancers18101579/s1, Figure S1: Effects of GGOH and FOH on cell lines; Figure S2: Flow cytometry gating strategy for MCC13; Figure S3: Flow cytometry gating strategy for MKL-1; Figure S4: Full uncropped Western blot images corresponding to Figure 7A with molecular weight markers and density ratios; Figure S5: Full uncropped Western blot images corresponding to Figure 7B with molecular weight markers and density ratios; Figure S6: Full uncropped Western blot images corresponding to Figure 8 with molecular weight markers and density ratios.

## Abbreviations

The following abbreviations are used in this manuscript:

MCC	Merkel cell carcinoma
VN-MCC	Viral-negative MCC
VP-MCC	Viral-positive MCC
MCPyV	Merkel cell polyomavirus
IBP	Isoprenoid biosynthesis pathway
HMGCR	HMG-CoA reductase
FPP	Farnesyl pyrophosphate
GGPP	Geranylgeranyl pyrophosphate
DGBP	Digeranyl bisphosphonate
FTI	Farnesyl transferase inhibitor
GGTI	Geranylgeranyl transferase inhibitor
GGDPS	Geranylgeranyl diphosphate synthase
MVA	Mevalonic acid
FOH	Farnesol
GGOH	Geranylgeraniol
