# Supplementary material for: Does Ras/Rho Have Skin in the Game: The Importance of the Isoprenoid Biosynthesis Pathway in Merkel Cell Carcinoma Cell Lines
Source: Cancers (Basel). 2026 May 13;18(10):1579. doi: 10.3390/cancers18101579 (PMC13205007; doi:10.3390/cancers18101579)
Supplement: Supplementary file 1 [file cancers-18-01579-s001.zip › cancers-4277950-supplementary.pdf]

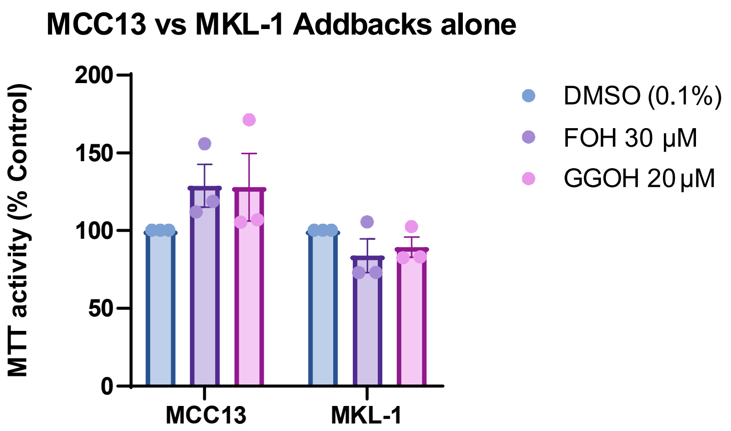

Supplemental Figure S1. FOH and GGOH addbacks across cell lines. MCC13 and MKL-1 cells were supplemented with FOH (30 μM) and GGOH (20 μM) alone and compared. Viability was measured by MTT assay. Cells are represented as a percent of the DMSO control.

A.

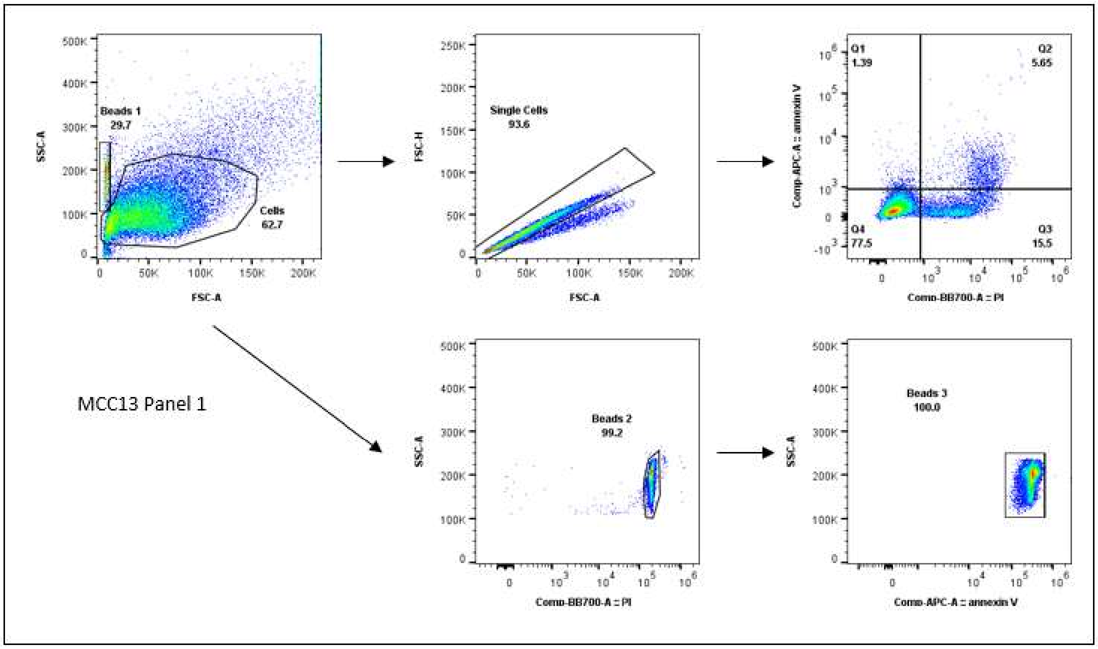

B.

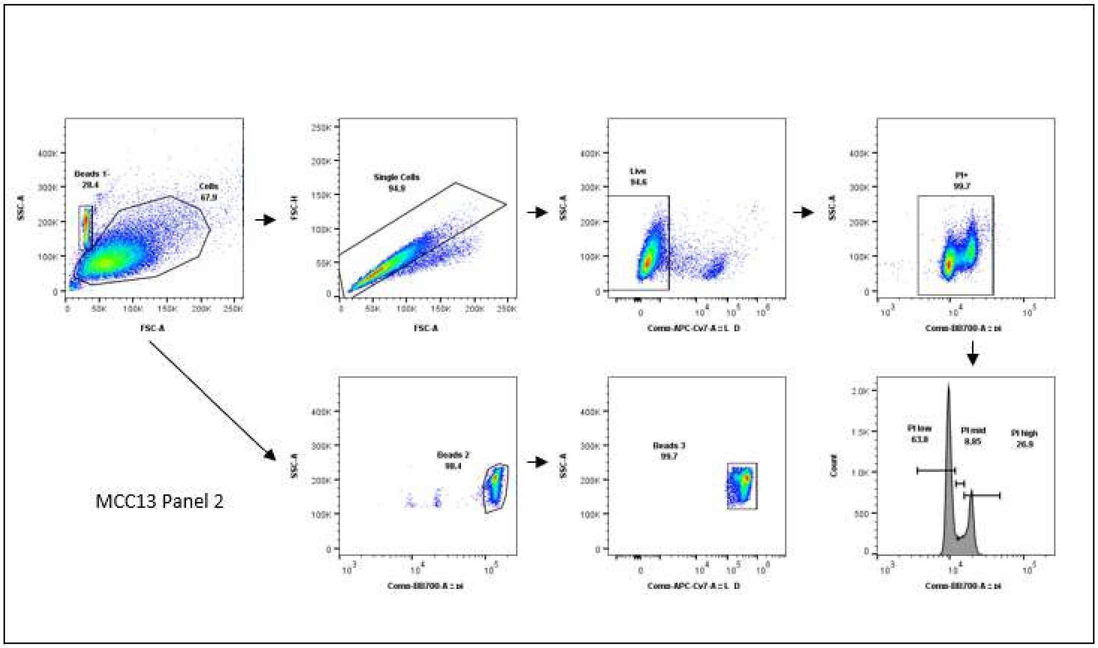

Supplemental Figure S2. Flow Cytometry Gating Schematic for MCC13. Gating strategies for panels 1 (A) and 2 (B) for the MCC13 tumor line. A) MCC13 tumor cells were gated from CountBright™ Absolute Counting Beads, followed by single cells, then PI and Annexin V. Quadrant percentages and absolute cell counts were calculated using FlowJo software. CountBright™ Absolute Counting Beads were isolated from MCC13 tumor cells and subsequently gated using available channels for bead purity. B) MCC13 tumor cells were gated from CountBright™ Absolute Counting Beads, followed by single cells, then viable cells followed by PI. PI⁺ peaks were divided into Low, Mid, and High expression and percentages and absolute cell count were calculated using FlowJo software. CountBright™ Absolute Counting Beads were isolated from MCC13 tumor cells and subsequently gated using available channels for bead purity.

A.

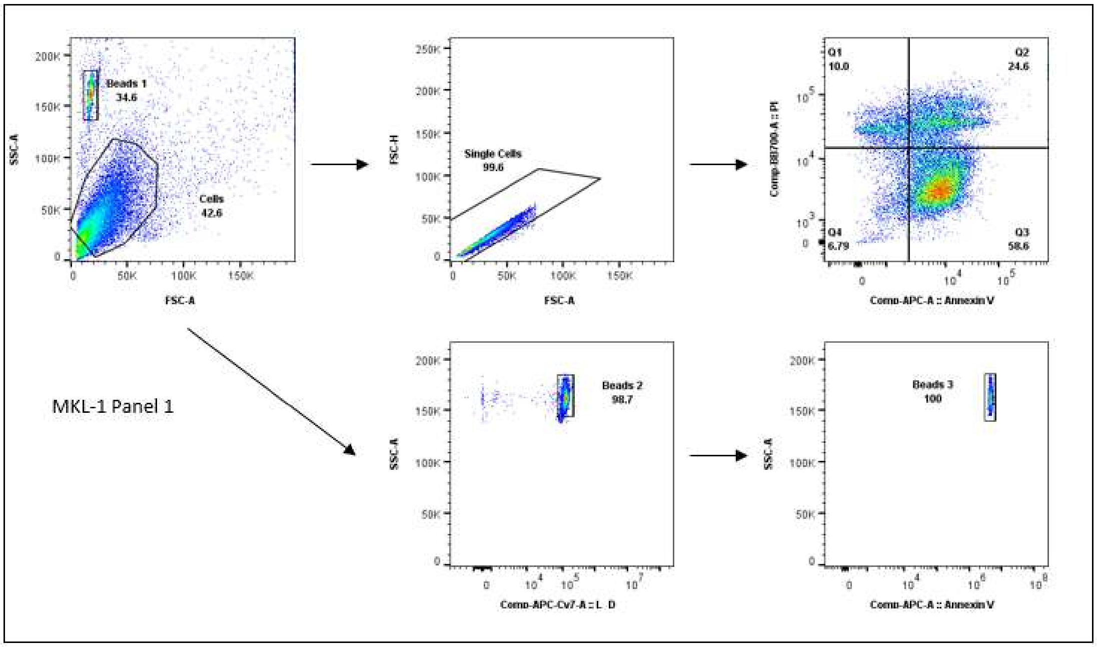

B.

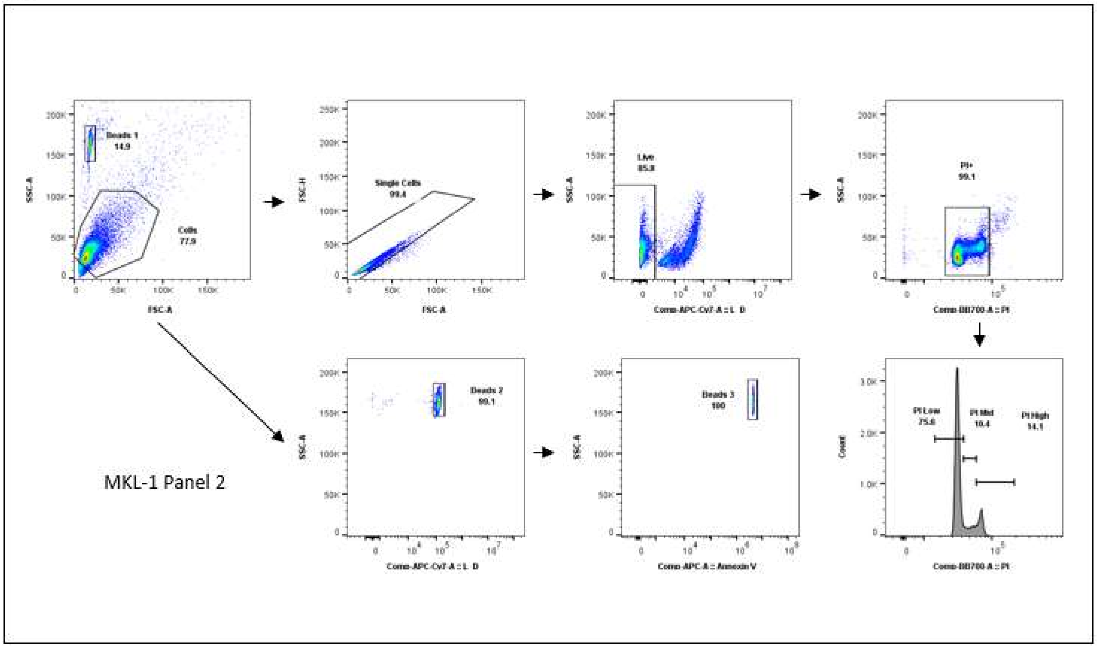

Supplemental Figure S3. Flow Cytometry Gating Schematic for MKL-1. Gating strategies for panels 1 (A) and 2 (B) for the MKL-1 tumor line. A) MKL-1 tumor cells were gated from CountBright™ Absolute Counting Beads, followed by single cells, then PI and Annexin V. Quadrant percentages and absolute cell counts were calculated using FlowJo software. CountBright™ Absolute Counting Beads were isolated from MKL-1 tumor cells and subsequently gated using available channels for bead purity. B) MKL-1 tumor cells were gated from CountBright™ Absolute Counting Beads, followed by single cells, then viable cells followed by PI. PI^{+} peaks were divided into Low, Mid, and High expression and percentages and absolute cell count were calculated using FlowJo software. CountBright™ Absolute Counting Beads were isolated from MKL-1 tumor cells and subsequently gated using available channels for bead purity.

S4

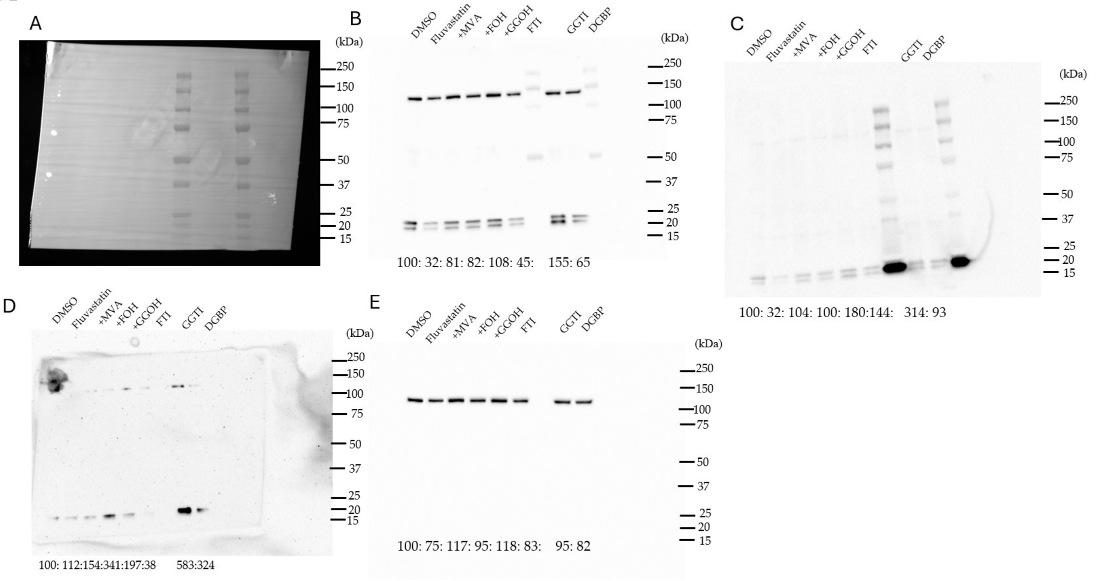

Supplemental Figure S4: Uncropped western blot images for Figure 7A with corresponding molecular weight ladders and density ratios. A) MCC13 colometric image of blot showing molecular weight ladder placement. B) MCC13 western blot probed for Cdc42. C) MCC13 western blot probed for Rac1. D) MCC13 western blot probed for RhoA. E) MCC13 western blot probed for house keeping gene, vinculin.

S5

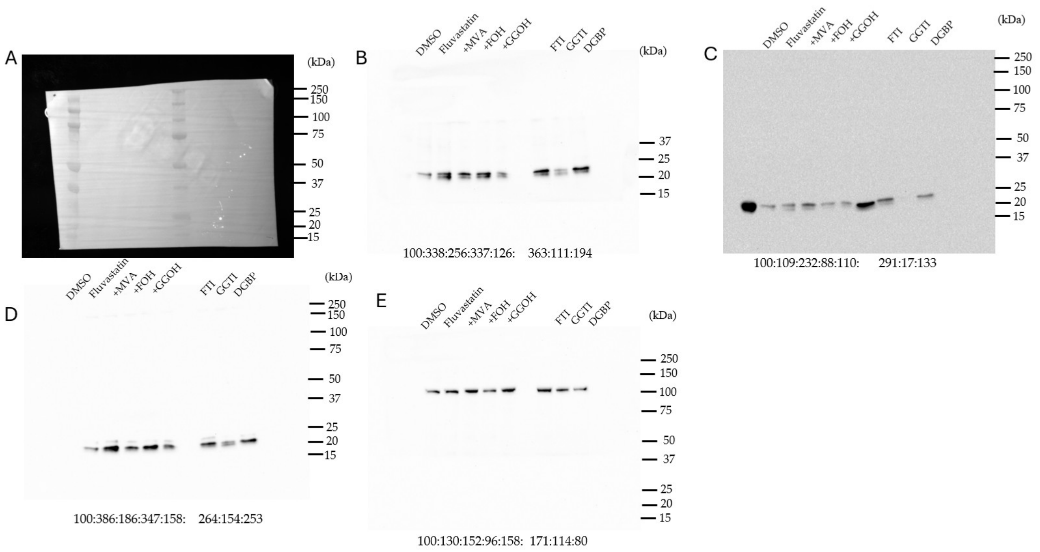

Supplemental Figure S5: Uncropped western blot images for Figure 7B with corresponding molecular weight ladders and density ratios. A) MKL-1 colometric image of blot showing molecular weight ladder placement. B) MKL-1 western blot probed for Cdc42. C) MKL-1 western blot probed for Rac1. D) MKL-1 western blot probed for RhoA. E) MKL-1 western blot probed for house keeping gene, vinculin.

S6

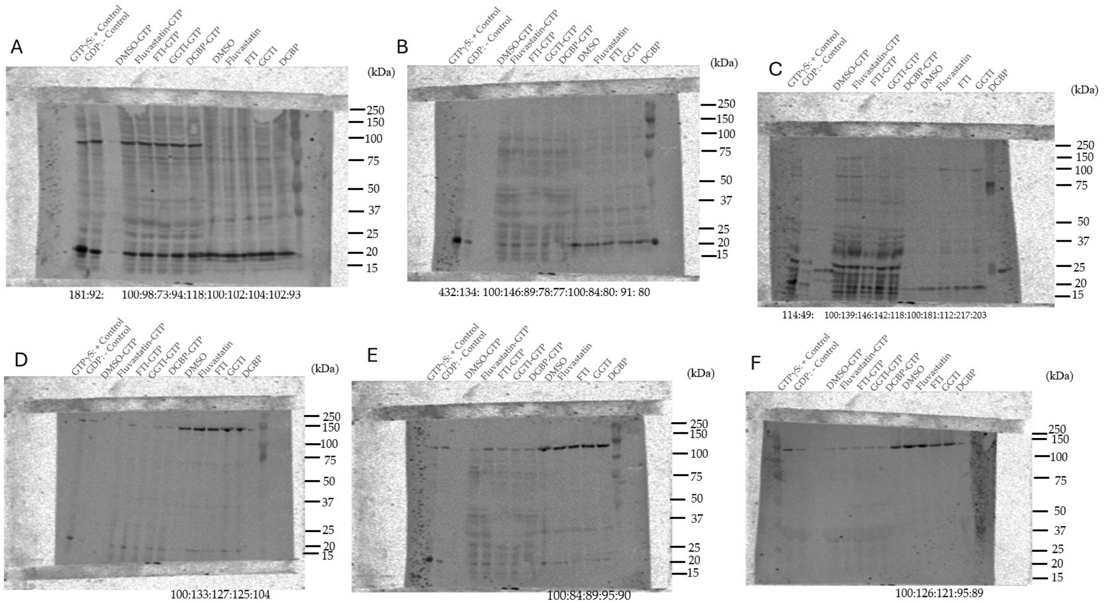

Supplemental Figure S6: Uncropped western blot images for Figure 8 with corresponding molecular weight ladders and density ratios. A) MKL-1 GTPase pull down for Cdc42. B) MKL-1 GTPase pull down for Rac1 C) MKL-1 GTPase pull down for RhoA. D) Cdc42 blot probed for vinculin E) Rac1 blot probed for vinculin F) RhoA blot probed for vinculin
